# Photobiomodulation Promotes Repair Following Spinal Cord Injury by Regulating the Transformation of A1/A2 Reactive Astrocytes

**DOI:** 10.3389/fnins.2021.768262

**Published:** 2021-11-02

**Authors:** Xuankang Wang, Zhihao Zhang, Zhijie Zhu, Zhuowen Liang, Xiaoshuang Zuo, Cheng Ju, Zhiwen Song, Xin Li, Xueyu Hu, Zhe Wang

**Affiliations:** ^1^Department of Orthopedics, Xijing Hospital, Fourth Military Medical University, Xi’an, China; ^2^Hospital of People’s Liberation Army Joint Logistic Support Force, Dalian, China

**Keywords:** spinal cord injury, photobiomodulation, A1/A2 astrocytes, basic fibroblast growth factor, transforming growth factor-β

## Abstract

After spinal cord injury (SCI), reactive astrocytes can be classified into two distinctive phenotypes according to their different functions: neurotoxic (A1) astrocytes and neuroprotective (A2) astrocytes. Our previous studies proved that photobiomodulation (PBM) can promote motor function recovery and improve tissue repair after SCI, but little is known about the underlying mechanism. Therefore, we aimed to investigate whether PBM contributes to repair after SCI by regulating the activation of astrocytes. Male rats subjected to clip-compression SCI were treated with PBM for two consecutive weeks, and the results showed that recovery of motor function was improved, the lesion cavity size was reduced, and the number of neurons retained was increased. We determined the time course of A1/A2 astrocyte activation after SCI by RNA sequencing (RNA-Seq) and verified that PBM inhibited A1 astrocyte activation and promoted A2 astrocyte activation at 7 days postinjury (dpi) and 14 dpi. Subsequently, potential signaling pathways related to A1/A2 astrocyte activation were identified by GO function analysis and KEGG pathway analysis and then studied in animal experiments and preliminarily analyzed in cultured astrocytes. Next, we observed that the expression of basic fibroblast growth factor (bFGF) and transforming growth factor-β (TGF-β) was upregulated by PBM and that both factors contributed to the transformation of A1/A2 astrocytes in a dose-dependent manner. Finally, we found that PBM reduced the neurotoxicity of A1 astrocytes to dorsal root ganglion (DRG) neurons. In conclusion, PBM can promote better recovery after SCI, which may be related to the transformation of A1/A2 reactive astrocytes.

## Introduction

The incidence of spinal cord injury (SCI) has gradually increased in recent years, imposing a great burden on individuals, families, and society, but there is no effective intervention for SCI (SPI, 2019). SCI leads to degeneration of cells, including neurons, microglia, astrocytes, and oligodendrocytes, in the epicenter of the lesion. Following the primary injury caused by physical impact, secondary damage (including neuroinflammation, excitotoxicity, apoptosis, and oxidative stress) delays tissue repair and causes additional loss of neurons and glial cells, hindering tissue repair (O’Shea et al., 2017). As one of the main types of innate immune cells, astrocytes can be divided adopt a neurotoxic (A1) or neuroprotective (A2) phenotype, are activated from a homeostatic state to a reactive state by different neuropathological stimuli, similar to microglia/macrophages (M1/M2) (Liddelow et al., 2017). A1 astrocytes can cause the death of neurons and oligodendrocytes, can destroy synapses, and are induced by neuroinflammation; A2 astrocytes have a neuroprotective effect, tend to promote tissue repair and regeneration, and are activated by ischemia (Liddelow et al., 2017). A1 and A2 astrocytes express different transcripts, which may explain the differences in the roles they may play in the course of diseases or injuries (Kwon and Koh, 2020; Li X. et al., 2020). Therefore, the regulation of A1/A2 astrocyte activation has been regarded as a target for the treatment of central nervous system (CNS) diseases or injuries in recent studies (Xu et al., 2018; Liu et al., 2019, 2020; Vismara et al., 2020; Zheng et al., 2021).

Photobiomodulation (PBM), or the use of low levels of red or near-infrared light (NIR), has already been applied to reduce inflammation, regulate the immune system, and promote wound healing and tissue regeneration (Rojas and Gonzalez-Lima, 2013). Recently, increasing interest has been paid to its application in dementia, Parkinson’s disease (PD), stroke, brain trauma, and SCI due to its widespread and mild biological effect (Salehpour et al., 2018). Our team previously found that 810 nm PBM can regulate macrophage/microglial polarization, reduce secondary inflammation, inhibit scar formation, and promote the recovery of motor function after SCI (Song et al., 2017; Sun et al., 2020; Zhang et al., 2020). However, little is known about the underlying mechanism, limiting its further application.

According to recent reports, some classic signaling pathways associated with the inflammatory response and immune system processes participate in A1/A2 astrocyte activation in various contexts (Xu et al., 2018; Hensel et al., 2019; Qian et al., 2019; Rakers et al., 2019; Zou et al., 2019; Li T. et al., 2020; Liu et al., 2020; Ma et al., 2020; Wang et al., 2020; Zheng et al., 2021). Therefore, it is necessary to explore the mechanism of A1/A2 astrocyte activation after SCI due to the complexity of CNS pathology. We identified these pathways by KEGG pathway analysis, and the roles of the nuclear factor-kappa B (NF-κB) pathway (Liu et al., 2020; Zheng et al., 2021), Notch pathway (Qian et al., 2019), Janus kinase 2 (JAK2)–signal transducer and activator of transcription 3 (STAT3) pathway (Rakers et al., 2019; Ma et al., 2020; Wang et al., 2020), and PI3K–Akt pathway (Xu et al., 2018; Li T. et al., 2020) in the effect of PBM treatment were further assessed. We suspect that PBM, in addition to modulating signaling pathways, can also change the levels of some inflammation-related factors or neuroprotective factors to indirectly regulate A1/A2 astrocyte activation. Basic fibroblast growth factor (bFGF) and transforming growth factor-β (TGF-β) were identified as targets because both were stimulated by PBM treatment *in vivo* and *in vitro*. As multifunctional growth factors, bFGF and TGF-β have been proven to be involved in the development, proliferation, and morphological transformation of astrocytes, and functional changes in these cells (Chao et al., 1992; Agarwal and Bergles, 2014; Stork et al., 2014; Zhang et al., 2018). In this study, we proved that PBM can regulate astrocyte activation and contribute to phenotype transformation to promote repair after SCI. The mechanism may be associated with the regulation of signaling pathways that are key for astrocyte activation and indirect regulation *via* increases in the levels of bFGF and TGF-β.

## Materials and Methods

### Spinal Cord Injury Model

Male Sprague-Dawley rats (8–10 weeks) were obtained from the Animal Centre of Air Force Medical University. A modified bilateral compression SCI model was established as described previously (Song et al., 2017). The rats were anesthetized with 50 mg/kg pentobarbital sodium (by intraperitoneal injection) and then subjected to laminectomy at T10. SCI was induced by completely closing a forceps (0.5 mm width, Fine Science Tools) on the exposed spinal cord for 40 s. Manual urination was performed twice daily until reestablishment of the micturition reflex. Biocompatible laser fibers were embedded within the lamina on the top of the spinal cord in the SCI + vehicle group and SCI + PBM group. The sham group underwent the same surgical procedure except that the spinal cord was not compressed. The animal procedures were approved by the Institutional Animal Care and Use Committee of Air Force Medical University.

### Photobiomodulation Therapy

Rats were anesthetized (50 mg/kg pentobarbital sodium, intraperitoneal injection) and exposed to a low-level laser daily for PBM treatment. A continuous 810-nm diode laser beam (MW-GX-808, Lei Shi Optoelectronics Co., Ltd., Changchun, China) was used in animal models. The parameters are referred to in our previous research (Song et al., 2017; Liang et al., 2019), more details are illustrated in Supplementary Table 1. PBM treatment was administered for 14 consecutive days, and the sham group and SCI + vehicle group received the same treatments except for PBM.

### Behavioral Analysis

The Basso–Beattie–Bresnahan (BBB) scale (Basso et al., 1995) was used to assess functional recovery before surgery and at 1–, 3–, 5–, 7–, 14–, 21–, and 28-days postinjury (dpi). A score of 0 indicates complete paralysis, and a score of 21 indicates complete mobility. The Louisville Swimming Scale (LSS) (Smith et al., 2006) was used to evaluate the alternation and movement of the hindlimbs and the dependence of the forelimbs, the angle of the body and the instability of the trunk before surgery and at 3–, 7–, 14–, and 28-dpi. For gait analysis, the rats were required to traverse a straight runway lined with white paper (Ma et al., 2001). The hindlimbs (blue) and hindlimbs (red) were dyed with non-toxic and non-irritating ink, and then the step width was analyzed. The results were analyzed by two observers blinded.

### Tissue Processing

Rats were anesthetized and perfused intracardially with 4% paraformaldehyde (PFA), and an approximately 2-cm segment containing an equal length of normal spinal cord tissue from the areas rostral and caudal to the epicenter was carefully dissected from the lesion site. The spinal cord was incubated in 4% PFA for 6 h and then dehydrated in 25% glucose phosphate buffer at 4°C until it sank. Next, the tissue was placed face up on a specimen chuck and embedded in OCT embedding agent, and the specimen chuck was placed on the quick-freezing table of a cryostat (CM1900, Leica) for quick freezing and embedding. When the OCT became white and hard, the tissue was sectioned. Approximately 10-μm-thick serial sagittal sections were cut and then placed on slides. Sections from the area near the epicenter were selected for each subject, stored at −20°C and were prepared for immunofluorescence.

Rats were perfused with saline, and an approximately 1-cm spinal cord segment from the lesion site was collected, snap frozen in liquid nitrogen, and stored at −80°C. The samples were prepared for RNA sequencing (RNA-Seq), real-time PCR (RT-PCR), and western blot analysis.

### Immunofluorescence

Frozen sections were rinsed in phosphate-buffered saline (PBS) and then blocked in PBS containing 0.3% Triton X-100 and 1% donkey serum for 30 min. The sections were incubated with the primary antibodies at 4°C overnight. The following primary antibodies were used: anti-NeuN (1:100, ab177487, Abcam), anti-GFAP (1:400, ab4674, Abcam), anti-C3 (1:300, ab200999, Abcam), anti-S100a10 (1:200, PA5-95505, Invitrogen), and anti-beta III tubulin (1:400, ab78078, Abcam). After washing with PBS, the sections were incubated with appropriate secondary antibodies at 37°C for 2 h and counterstained with 4′,6-diamidino-2-phenylindole (DAPI) the next day. Fluorescence images were acquired with a fluorescence microscope (BX51, Olympus). Three non-consecutive sections (imaged using a 20× objective lens) from each subject and five separate visual fields from each section were randomly selected and analyzed. All images were analyzed using ImageJ (NIH), and all analyses were performed in a blinded manner.

### RNA Sequencing Analysis

As previously described (Li Y. C. et al., 2020), total RNA was extracted using TRIzol Reagent (Invitrogen), and the RNA concentration was assessed with an RNA 6000 Nano LabChip Kit (Agilent). An Epicenter Ribo-Zero Gold Kit (Illumina) was used to remove ribosomal RNA from 10 μg of total RNA. The RNA fraction was isolated after purification, and then a cDNA library was created according to the instructions of an mRNA-Seq sample preparation kit (Illumina). Libraries with cDNA target fragments of 250–350 bp were selected. HiSeq 4000 (Illumina) was used for paired-end sequencing, and subsequent sequencing experiments and bioinformatics analyses were performed at LC Bio (China).

### Real-Time PCR

RNA was extracted using an RNA extraction kit (Takara Bio), and subsequently reverse transcribed into cDNA using Evo M-MLV RT Premix (Accurate Biotechnology). The reaction conditions were 37°C for 15 min, 85°C for 5 s, and 4°C for 10 min, and a 10-μL solution containing cDNA (2 μL), ddH_2_O (2 μL), primers (1 μL), and SYBR Green (5 μL; Accurate Biotechnology) was prepared for RT-PCR according to the manufacturer’s instructions. The sequences of the primers used are provided in Table 1. GAPDH was used as an internal control, and the data were calculated by the 2^–ΔΔ^ ^CT^ method.

**TABLE 1:** Primer sequences used for RT-PCR.

**Gene**	**Forward primer sequence (5**′**–3**′)	**Reverse primer sequence (5**′**3**′)
Vim	GAGGAGATGAGGGAGTTGCG	CTGCAATTTTTCTCGCAGCC
Timp1	CGCTAGAGCAGATACCACGA	CCAGGTCCGAGTTGCAGAAA
Gfap	AACCGCATCACCATTCCTGT	TCCTTAATGACCTCGCCATCC
Serping1	TGGCTCAGAGGCTAACTGGC	GAATCTGAGAAGGCTCTATCCCCA
Fkbp5	TGCAGTGTCGGCAGTTGTA T	GGGTCGCCCAAGTTAGAACA
Gbp2	TAAAGGTCCGAGGCCCAAAC	AACATATGTGGCTGGGCGAA
Emp1	ACCA TTGCCAACGTCTGGAT	TGGAACACGAAGACCACGAG
S100a10	GAAAGGGAGTTCCCTGGGTT	CCCACTTTTCCATCTCGGCA
Ptgs2	CTCAGCCATGCAGCAAATCC	GGGTGGGCTTCAGCAGTAAT
TNF-α	CCCTCACACTCAGATCATCTTCT	GCTACGACGTGGGCTACAG
IL-6	ATTGTATGAACAGCGATGATGCAC	CCAGGTAGAAACGGAACTCCAG
IL-1β	CCCTGAACTCAACTGTGAAATAGCA	CCCAAGTCAAGGGCTTGGAA
iNOS	TGGTGAGGGGACTGGACTTT	ATCCTGTGTTGTTGGGCTGG
LCN2	CCGACACTGACTACGACCAG	AATGCATTGGTCGGTGGGAA
BDNF	GTCGCACGGTCCCCATTG	ACCTGGTGGAACTCAGGGT
NGF	CTGGACTAAACTTCAGCATTC	TGTTGTTAATGTTCACCTCGC
GDNF	GGAGACCGGATCCGAGG	GCGCTTCGAGAAGCCTCT
bFGF	TCCAAGCAGAAGAGAGAGGAG	GGCGTTCAAAGAAGAAACAC
TGF-β	GAGAGCCCTGGATACCAACTACTG	GTGTGTCCAGGCTCCAAATGTAG
GAPDH	GAACATCATCCCTGCATCCA	CCAGTGAGCTTCCCGTTCA

### Western Blot Analysis

Protein was isolated from spinal cord tissue or cultured cells by homogenizing the samples using RIPA lysis buffer supplemented with protease and phosphatase inhibitor cocktail (Thermo Fisher). The protein concentration was determined by the BCA assay, and 20–30 μg of protein from each sample was separated by SDS-PAGE (10 or 12%) and transferred onto NC membranes. After blocking with 5% BSA for 1 h at room temperature, the membranes were incubated with the following specific primary antibodies overnight at 4°C: C3 (ab200999, Abcam, 1:1000), S100a10 (PA5-95505, Invitrogen, 1:1000), NF-κB (8242, Cell Signaling Technology, 1:1000), pNF-κB (3033, Cell Signaling Technology, 1:1000), Notch1 (3608, Cell Signaling Technology, 1:1000), JAK2 (3230, Cell Signaling Technology, 1:1000), pJAK2 (AF3022, Affinity, 1:1000), STAT3 (12640, Cell Signaling Technology, 1:1000), pSTAT3 (ab76315, Abcam, 1:1000), PI3K (60225-1-Ig, Proteintech, 1:1000), pPI3K (AF3241, Affinity, 1:1000), Akt (4691, Cell Signaling Technology, 1:1000), pAkt (4060, Cell Signaling Technology, 1:1000), and β-actin (66009-1-Ig, Proteintech, 1:1000). After washing with TBST, the membranes were incubated with corresponding secondary antibodies for 1 h and then developed with ECL-Plus Reagent (Millipore), and the bands were visualized using an Amersham Imager 600 (General Electric).

### Primary Cell Culture

As described previously (Wolfes et al., 2017), primary astrocytes were isolated from Sprague-Dawley rat neonates (P0–P2). Briefly, the cerebral cortex was carefully cleaned, and the samples were digested with trypsin (0.125%). The digested tissue was shaken gently for 20 min and then resuspended in DMEM-F12 supplemented with 10% FBS and 1% penicillin-streptomycin (Sigma-Aldrich). A single-cell suspension was obtained by filtration through 100-μm nylon mesh and plated in cell flasks precoated with poly-L-lysine (Sigma-Aldrich). After 12–14 days in culture, the flasks were shaken at 200 rpm for 24 h to remove microglia, the medium was changed, and the remaining astrocytes were digested and re-cultured for follow-up experiments.

Dorsal root ganglia (DRGs) were harvested as described previously (Zhang et al., 2020). Briefly, DRGs were quickly dissected from Sprague-Dawley rat neonates (P0–P2). The attached nerve roots were removed and then shredded and digested in a solution of trypsin (0.125%) and collagenase IV (0.1%). After 30 min, the digested DRGs were rinsed in DMEM-F12 supplemented with 20% FBS to stop the digestion and then centrifuged at 1000 rpm for 5 min. The DRG neurons were then resuspended in neurobasal medium supplemented with B27 and penicillin/streptomycin solution (Invitrogen). The DRG neurons were plated at a density of 50,000 cells/well.

### Cell Treatment

A1 astrocytes were induced with 30 ng/ml tumor necrosis factor-α (TNF-α; 8902SF, Cell Signaling Technology), 400 ng/ml complement component 1q (C1q; MBS 143105, MyBioSource), and 3 ng/ml interleukin-1α (IL-1α; i3901, Sigma) for 24 h. For the appropriate PBM parameters for irradiating cultured astrocytes, refer to our previous report (Zheng et al., 2021).

More details are illustrated in Supplementary Table 1. The first irradiation was conducted immediately after A1 astrocyte induction, and another irradiation was performed again after 12 h. Twenty-four hours after A1 astrocyte induction, the culture medium was harvested and used as astrocyte-conditioned medium (ACM). Half of the DRG neuron culture medium was replaced with ACM, and the DRG neurons were cultured for another 24 h to evaluate the effect of astrocytes on neurons.

To explore the effects of bFGF and TGF-β on astrocytes, recombinant bFGF (3339-FB, R&D Systems) and TGF-β (HST-TB3, Stemimmune) were added to the medium 24 h after A1 astrocyte induction. To inhibit the pathways related to astrocyte activation, the following antagonists were added 1 h before A1 astrocyte induction: PDTC (20 μM, S3633, Selleckchem), DAPT (20 μM, S2215, Selleckchem), AG490 (10 μM, S1143, Selleckchem), LY294002 (20 μM, S1150, Selleckchem), PD173074 (200 nM, S1264, Selleckchem), and vactosertib (100 μM, Selleckchem).

### Enzyme-Linked Immunosorbent Assay

The levels of bFGF and TGF-β in the astrocyte culture medium 24 h after A1 astrocyte induction were measured by enzyme-linked immunosorbent assay (ELISA) kits (Meimian Industrial) in accordance with the manufacturer’s protocols.

### Statistical Analysis

Experiments were independently conducted at least three times. The data are shown as the mean ± standard deviation (SD) and were analyzed by GraphPad Prism 8 software. One-way analysis of variance (ANOVA) followed by Bonferroni’s *post hoc* test was used to analyze differences between groups at a specific time point. Two-way repeated-measures ANOVA followed by Bonferroni’s *post hoc* test was used to analyze the differences between groups at different time points. A *p*-value < 0.05 was considered statistically significant.

## Results

### Photobiomodulation Promoted the Recovery of Motor Function, Reduced the Lesion Cavity Size, and Increased the Number of Surviving Neurons After Spinal Cord Injury

**FIGURE 1:**
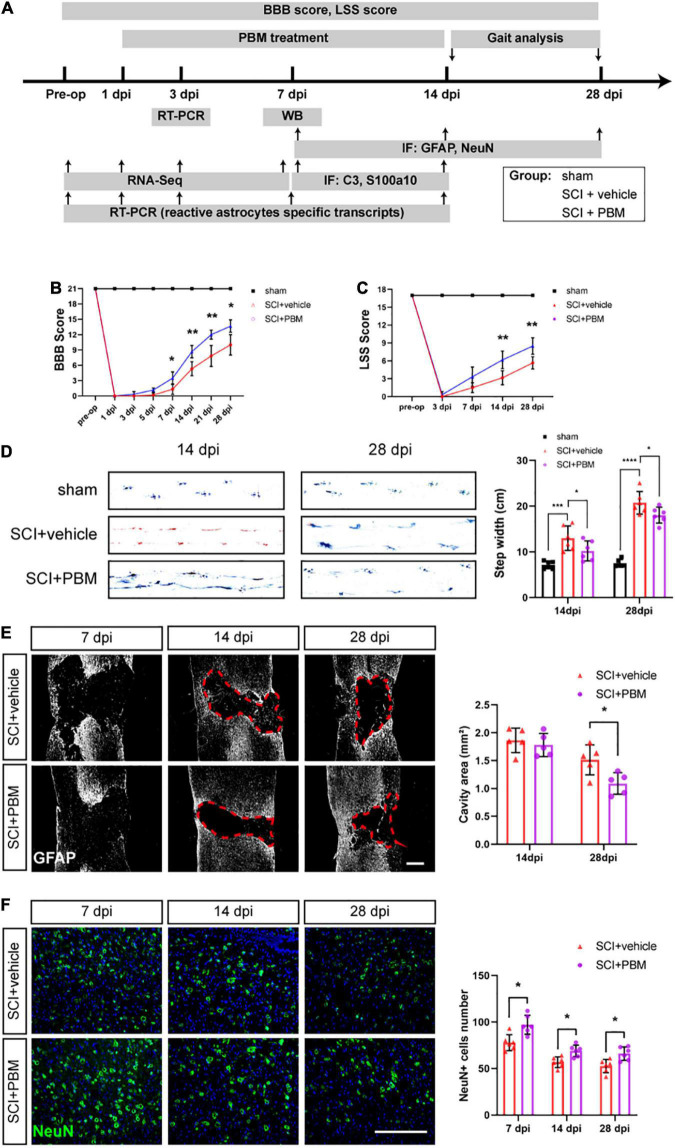
Photobiomodulation promoted motor recovery, reduced the lesion cavity size and increased the number of surviving neurons after SCI. **(A)** Timeline of the experimental design. **(B,C)** BBB scores and LSS scores were used to evaluate the motor function of each group (*n* = 6 individuals per group). **(D)** Representative footprints from each group at 14 and 28 dpi obtained by gait analysis. The average step width was calculated (*n* = 6 individuals per group). **(E)** Representative images of immunofluorescence staining of GFAP in spinal cord tissue from the SCI + vehicle and SCI + PBM groups at 7, 14, and 28 dpi. Quantification of the cavity area of spinal cord lesions at each time point (*n* = 5 individuals per group). Scale bar: 400 μm. **(F)** Representative images of immunofluorescence staining for NeuN in the ventral horn of the spinal cord within ± 150 μm from the lesion epicenter. Quantification of the number of NeuN^+^ cells in the SCI + vehicle group and SCI + PBM group at each time point (*n* = 6 individuals per group). Scale bar: 200 μm. *p < 0.05, **p < 0.01, ***p < 0.001.

Figure 1A is the experimental design in this study. To assess the motor function of the different groups after PBM, the BBB scale, the LSS, and gait analysis were used. According to BBB scores, PBM treatment improved motor function after SCI (SCI + PBM group vs. SCI + vehicle group: 0.33 ± 0.52 vs. 0.00 ± 0.00, 3 dpi; 1.17 ± 0.41 vs. 0.17 ± 0.41, 5 dpi; 3.50 ± 1.23 vs. 1.33 ± 1.03, 7 dpi; 8.67 ± 1.21 vs. 5.33 ± 1.37, 14 dpi; 12.00 ± 0.89 vs. 7.83 ± 2.04, 21 dpi; and 13.67 ± 1.21 vs. 10.00 ± 2.00, 28 dpi; Figure 1B). LSS scores were similar between the SCI + PBM group and the SCI + vehicle group (0.33 ± 0.52 vs. 0.00 ± 0.00, 3 dpi; 3.33 ± 1.63 vs. 1.50 ± 0.84, 7 dpi; 6.17 ± 1.47 vs. 3.17 ± 1.17, 14 dpi; and 8.50 ± 1.38 vs. 5.67 ± 1.03, 28 dpi; Figure 1C). Furthermore, gait analysis suggested that the step width of the SCI + PBM group was narrower than that of the SCI + vehicle group at 14 and 28 dpi (Figure 1D).

We next examined the effect of PBM on tissue repair after SCI. In response to secondary damage caused by SCI, the glial scar, which protects the injured site in the acute phase of SCI but prevents axonal regeneration in the subacute phase, gradually forms (Bradbury and Burnside, 2019). At 7 dpi, we did not observe a dense glial scar with well-defined margins. At 14 dpi, a glial scar with well-defined margins was present, and PBM had no obvious effect on the cavity area in any of the groups. At 28 dpi, the lesion area in the SCI + PBM group was smaller than that in the SCI + vehicle group (Figure 1E). We also counted the number of surviving motor neurons in the ventral spinal cord, which are essential for motor function, and the results showed that there were more neurons in the SCI + PBM group at 7, 14, and 28 dpi (Figure 1F).

### Analysis of Astrocyte-Specific Transcript Levels After Spinal Cord Injury

**FIGURE 2:**
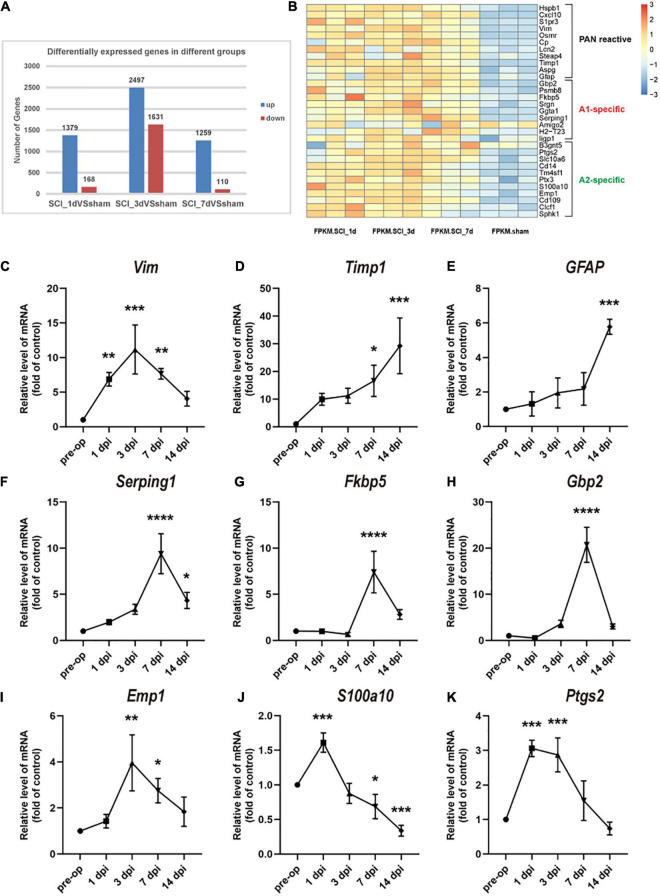
Changes in astrocyte-specific transcript levels after SCI. **(A)** The number of differentially expressed genes in the control, SCI-1d, SCI-3d, and SCI-7d groups. **(B)** A heatmap of selected significantly changed PAN-reactive, A1-specific, and A2-specific genes. **(C–E)** Fold changes in the levels of the representative PAN-reactive transcripts (Vim, Timp1, and GFAP) at each time point after SCI. **(F–H)** Fold changes in the levels of the representative A1-specific reactive transcripts (Serping1, Fkbp5, and Gbp2) at each time point after SCI. **(I–K)** Fold changes in the levels of the representative A2-specific transcripts (Emp1, S100a10, and Ptgs2) at each time point after SCI. *N* = 3 individuals per group. *p < 0.05, **p < 0.01, ***p < 0.001, ****p < 0.0001 vs. the sham control group.

To understand the changes in reactive astrocyte-specific transcript levels after SCI, we performed RNA-Seq experiments on spinal cord tissue. A total of 1379, 2497, and 1259 genes were upregulated and 168, 1631, and 110 genes were downregulated in the SCI group compared with the sham group at 1, 3, and 7 dpi, respectively (Figure 2A). Significantly changed astrocyte-specific transcripts were selected and classified as PAN-reactive, A1-specific, and A2-specific (Figure 2B) according to a previous report (Liddelow et al., 2017). Then, we selected representative genes for verification by RT-PCR. As indicated by the data, the levels of PAN-reactive transcripts (Vim, Timp1, and GFAP; Figures 2C–E) were highly upregulated generally within 14 dpi (except for Vim); the levels of A1-specific transcripts (Serping1, Fkbp5, and Gbp2; Figures 2F–H) reached a peak at 7 dpi; and the levels of A2-specific transcripts (Emp1, S100a10, and Ptgs2; Figures 2I–K) were generally first upregulated at 1 dpi (or at 3 dpi) and then downregulated at later time points. We conclude that astrocytes transform from a resting state to a reactive state in the acute phase after SCI. Specifically, astrocytes are polarized to the A1 phenotype beginning at 7 dpi. However, A2 polarization occurs earlier but to a lower degree than A1 polarization and then gradually fades.

### Photobiomodulation Modulated the Transformation of A1/A2 Astrocytes

**FIGURE 3:**
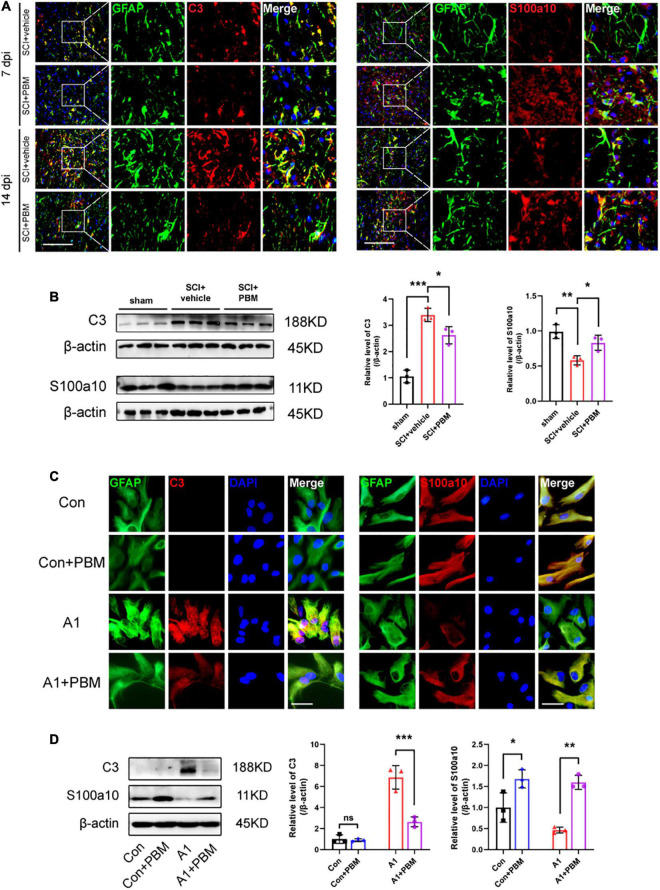
Photobiomodulation modulated the transformation of A1/A2 astrocytes. **(A)** Representative images of immunofluorescence staining for GFAP (green) and C3 (red) or S100a10 (red) in the lesion area 150 μm from the epicenter in the SCI + vehicle and SCI + PBM groups at 7 and 14 dpi. Scale bar: 200 μm. **(B)** Western blot analysis and quantification of the expression levels of C3 and S100a10 in each group at 7 dpi (*n* = 3 individuals per group). **(C)** Representative immunofluorescence images of GFAP (green) and C3 (red) or S100a10 (red) in astrocytes in the control, control + PBM, A1, and A1 + PBM groups. Con, control; A1, A1 astrocytes. Scale bar: 200 mm. **(D)** Representative blots and quantification of the expression levels of C3 and S100a10 in each group. The experiments were independently repeated three times. *p < 0.05, **p < 0.01, ***p < 0.001.

Subsequently, we examined the level of astrocyte activation in the SCI + vehicle group and SCI + PBM group. GFAP-positive and C3-positive cells were considered A1 astrocytes, and GFAP-positive and S100a10-positive cells were considered A2 astrocytes (Liddelow et al., 2017). We observed that A1 astrocyte activation began in the area around the epicenter at 7 dpi and that the proportion of A1 astrocytes was further increased at 14 dpi (Figure 3A). A1 astrocyte activation was significantly inhibited in the SCI + PBM group compared with the SCI + vehicle group at both 7 and 14 dpi, while A2 astrocyte activation was promoted by PBM treatment. The western blot results also proved that the upregulation of C3 expression and downregulation of S100a10 expression at 7 dpi were reversed by PBM (Figure 3B).

Next, we isolated astrocytes from rat neonates and induced their polarization to the A1 phenotype *in vitro* to further explore the effect of PBM on astrocytes in both a homeostatic state and an activated state. The expression of C3 in homeostatic astrocytes was too low to detect regardless of whether PBM was applied. A1 astrocytes were subjected to PBM, and the significant increase in the expression of C3 was inhibited. Homeostatic astrocytes expressed S100a10, and S100a10 expression was downregulated in A1-induced astrocytes. PBM increased S100a10 expression in astrocytes both in a homeostatic state and in an activated state (Figures 3C,D). Therefore, PBM had an effect on astrocytes in both an SCI model and under culture conditions.

### Signaling Pathways Involved in A1/A2 Astrocyte Activation Were Regulated by Photobiomodulation

**FIGURE 4:**
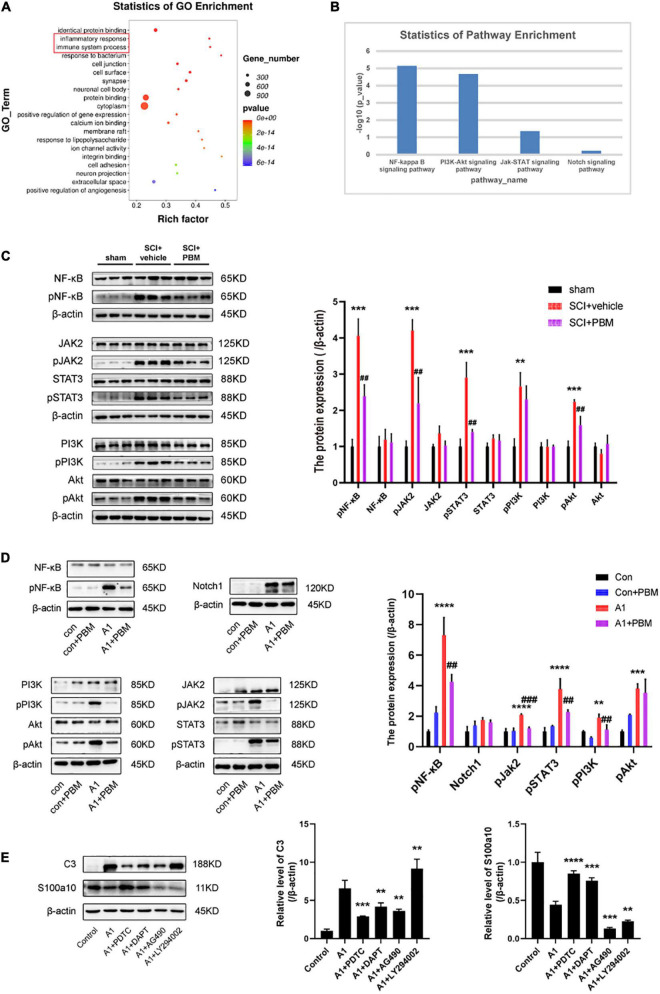
Signaling pathways involved in A1/A2 astrocyte activation were regulated by PBM. **(A)** GO function analysis between the SCI-7d group and sham control group. The inflammatory response and immune system process were dramatically activated. **(B)** The histogram shows the –log10 (*p*-value) of the NF-κB pathway, PI3K–Akt pathway, JAK–STAT pathway, and Notch pathway (comparison between multiple groups vs. the sham control group), as determined by KEGG enrichment analysis. **(C)** Western blot analysis of the expression of proteins related to the NF-κB signaling pathway, JAK2–STAT3 signaling pathway, and PI3K–Akt signaling pathway in the sham control, SCI + vehicle, and SCI + PBM groups at 7 dpi. Quantification of the relative protein levels of pNF-κB, NF-κB, pJAK2, JAK2, pSTAT3, STAT3, pPI3K, PI3K, pAkt, and Akt. *n* = 3 individuals per group. **p < 0.01, ***p < 0.001, the SCI + vehicle group vs. the sham control group; ^##^*p* < 0.01, the SCI + PBM group vs. the SCI + vehicle group. **(D)** Representative blots and quantification of the relative levels of pNF-κB, NF-κB, Notch1, pJAK2, JAK2, pSTAT3, STAT3, pPI3K, PI3K, pAkt, and Akt in the control, control + PBM, A1, and A1 + PBM groups. Con, control; A1, A1 astrocytes. The experiments were independently repeated three times. **p < 0.01, ***p < 0.001, ****p < 0.0001, the A1 group vs. the control group; ^##^*p* < 0.01, ^###^*p* < 0.001, the A1 + PBM group vs. the A1 group. **(E)** Representative blots and quantification of the relative levels of C3 and S100a10 in astrocytes treated with different inhibitors. The experiments were independently repeated three times. **p < 0.01, ***p < 0.001, ****p < 0.0001 vs. the A1 group.

To better understand the mechanism underlying A1/A2 astrocyte activation and the effect of PBM, GO biological process enrichment analysis and KEGG pathway enrichment analysis were performed. The results showed that the inflammatory response and immune system processes were both markedly altered at 7 dpi after SCI (Figure 4A). Previous studies have shown that the NF-κB pathway (Liu et al., 2020; Zheng et al., 2021), Notch pathway (Qian et al., 2019), JAK2–STAT3 pathway (Rakers et al., 2019; Ma et al., 2020; Wang et al., 2020), fibroblast growth factor (FGF) pathway (Hensel et al., 2019; Zou et al., 2019), and PI3K–Akt pathway (Xu et al., 2018; Li T. et al., 2020) are classic signaling pathways associated with inflammatory and immune responses related to A1/A2 astrocyte activation in different CNS disease and injury models. Next, we examined the candidate pathways identified by KEGG pathway enrichment. The analysis revealed that the NF-κB pathway, PI3K–Akt pathway, and JAK–STAT pathway were obviously activated in the SCI groups compared with the sham groups at 1, 3, and 7 dpi; however, the Notch pathway was not significantly changed and the FGF pathway was not identified by KEGG pathway enrichment analysis (Figure 4B). Therefore, we suspect that the activation of astrocytes in the acute phase after SCI may be related to these pathways, so we further examined the extent of pathway activation at 7 dpi. The western blot results suggested that these pathways were all activated after injury to some extent, and the expression of pNF-κB, pJAK2, pSTAT3, pPI3K, and pAkt was upregulated (Figure 4C). As expected, PBM had an anti-inflammatory effect, and the upregulation of the expression of the abovementioned activated molecules except pPI3K was inhibited by PBM for seven consecutive days.

To further verify that these pathways participate in the process by which PBM regulates astrocyte activation, we carried out experiments in culture. Astrocytes in a resting state and induced state were subjected to PBM. As expected, the expression of pNF-κB, Notch1, pJAK2, pSTAT3, pPI3K, and pAkt was upregulated in A1 astrocytes compared to astrocytes in a homeostatic state. In astrocytes in a homeostatic state, the expression of these molecules was not significantly different before and after PBM treatment. In A1 astrocytes, the increases in the levels of the key molecules pNF-κB, pJAK2, pSTAT3, pPI3K, and pAkt were suppressed by PBM, but there was no obvious change in the expression of Notch1 (Figure 4D). Next, we pretreated astrocytes with specific pathway antagonists, including PDTC (an NF-κB pathway inhibitor), DAPT (a Notch pathway inhibitor), AG490 (a JAK2–STAT3 pathway inhibitor), and LY294002 (a PI3K–Akt pathway inhibitor) before inducing their polarization to the A1 phenotype. Interestingly, when the NF-κB pathway and Notch pathway were inhibited, A1 astrocyte activation was blocked and A2 astrocyte activation was promoted, and when the JAK2–STAT3 pathway was inhibited, A1 astrocyte activation and A2 astrocyte activation were both hindered; however, when the PI3K–Akt pathway was inhibited, A2 astrocyte activation was blocked and A1 astrocyte activation was promoted (Figure 4E).

### Basic Fibroblast Growth Factor and Transforming Growth Factor-β Expression Was Upregulated by Photobiomodulation and Inhibited by A1 Astrocyte Activation

**FIGURE 5:**
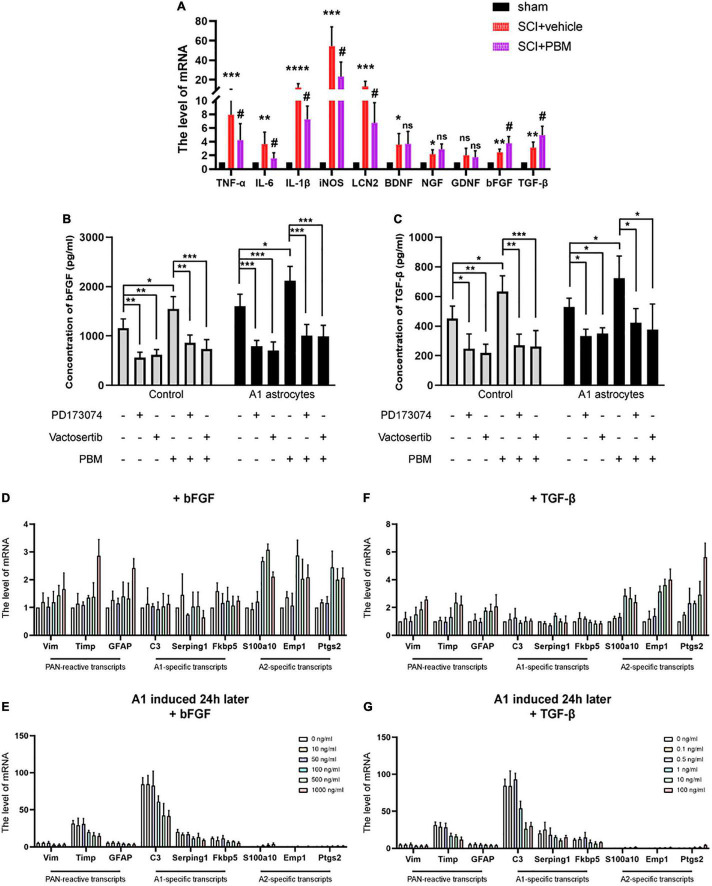
Basic fibroblast growth factor and TGF-β expression was upregulated by PBM and inhibited by A1 astrocyte activation. **(A)** The mRNA levels of proinflammatory factors and neurotrophic factors in each group at 3 dpi. *n* = 5 individuals per group. *p < 0.05, **p < 0.01, ***p < 0.001, ****p < 0.0001, the SCI + vehicle group vs. the sham control group; ^#^*p* < 0.05, ns, not significant, the SCI + PBM group vs. the SCI + vehicle group. **(B,C)** The levels of bFGF and TGF-β in the indicated medium were measured by ELISA. The experiments were independently repeated four times. *p < 0.05, **p < 0.01, ***p < 0.001. **(D,F)** The effect of different concentrations of bFGF and TGF-β on reactive astrocyte-related transcript levels in resting astrocytes. **(E,G)** The effects of different concentrations of bFGF and TGF-β on reactive astrocyte-related transcript levels in A1 astrocytes 24 h after induction. The data are shown as the fold change compared to the control group, as determined by RT-PCR. The experiments were independently repeated three times.

According to our previous studies, PBM can suppress the high expression of proinflammatory factors and promote the expression of neurotrophic factors after SCI (Song et al., 2017; Zhang et al., 2020). We suspect that there may be mechanisms by which PBM upregulates or downregulates the expression of one or several cytokines to indirectly exert a biological effect on astrocytes. Therefore, we evaluated the changes in the expression of some classic proinflammatory factors [TNF-α, interleukin-6 (IL-6), interleukin-1β (IL-1β), inducible nitric oxide synthase (iNOS), and lipocalin 2 (LCN2)] and some classic neurotrophic factors [brain-derived neurotrophic factor (BDNF), nerve growth factor (NGF), glial cell line-derived neurotrophic factor (GDNF), bFGF, and TGF-β] at 3 dpi by RT-PCR. The high expression of proinflammatory factors after SCI was downregulated by PBM, and the expression of bFGF and TGF-β was further upregulated by PBM; however, the levels of the other neurotrophic factors showed no obvious change (Figure 5A). Considering that A1 astrocyte activation is induced by inflammatory mediators themselves (C1q, TNF-α, and IL-1α) (Liddelow et al., 2017), we focused on bFGF and TGF-β, which may be related to the mechanism by which PBM inhibits A1 astrocyte activation. First, we observed that PBM increased the expression of bFGF and TGF-β in the culture medium of astrocytes in a homeostatic state and A1 astrocytes. Then, we assessed the ability of antagonists of bFGF (PD173074) and TGF-β (vactosertib) to inhibit the upregulation of FGF and TGF-β expression. PD173074 and vactosertib inhibited the secretion of bFGF and TGF-β and counteracted the effect of PBM in promoting the secretion of bFGF and TGF-β (Figures 5B,C). This finding also suggests that FGF- and TGF-β-related pathways may intersect and that PBM can act on one to affect the other.

Next, we tested the effect of different concentrations of recombinant bFGF and TGF-β proteins on astrocytes. We found that at a relatively lower concentration, bFGF and TGF-β had basically no influence on reactive astrocyte-related transcript levels in astrocytes in a homeostatic state; when a certain concentration was exceeded (bFGF: 100 ng/ml; TGF-β: 1 ng/ml), A2-specific transcript levels were significantly increased, but PAN-reactive transcript levels and A1-specific transcript levels were changed when the concentrations were higher (Figures 5D,E). Hereafter, we added the same concentrations of bFGF and TGF-β to A1 astrocytes after 24 h. Similarly, we observed that PAN-reactive transcript levels and A1-specific transcript levels were decreased at certain concentrations of bFGF and TGF-β, while A2-specific transcript levels were increased (Figures 5F,G). In conclusion, these data show that upregulation of bFGF and TGF-β expression contributes to the mechanism by which PBM promotes the transformation of A1 astrocytes to A2 astrocytes.

### Photobiomodulation Alleviated the Neurotoxic Effect of A1 Astrocytes on Dorsal Root Ganglion Neurons

**FIGURE 6:**
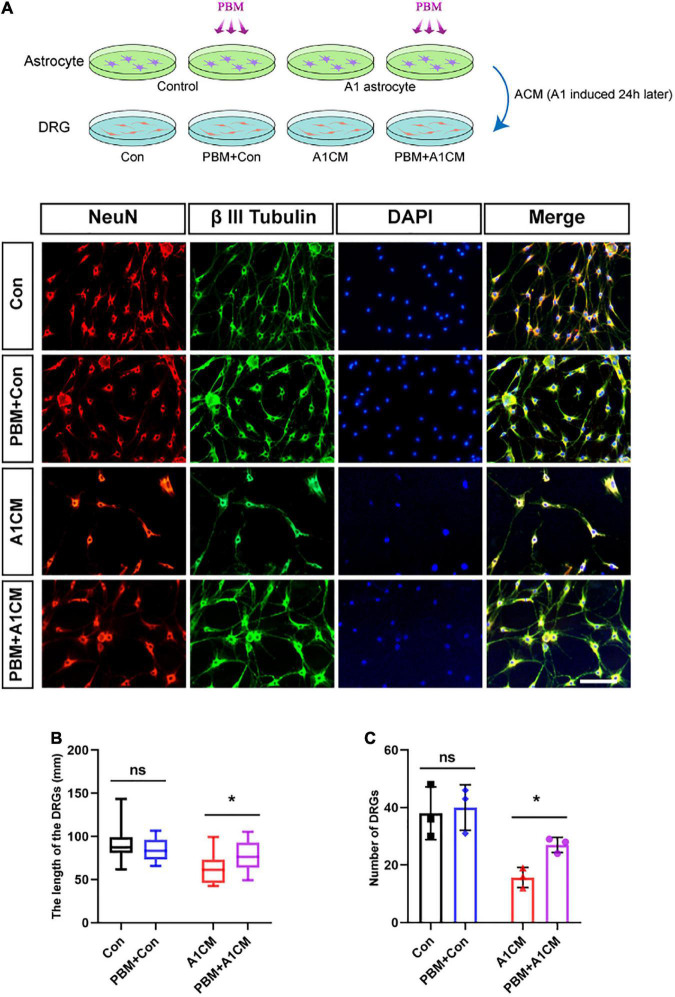
Photobiomodulation alleviated the neurotoxic effect of A1 astrocytes on DRG neurons. **(A)** DRG neurons treated with ACM collected 24 h after different treatments. Con, medium from control resting astrocytes; Con + PBM: medium from resting astrocytes treated with PBM; A1CM, medium from A1 astrocytes; PBM + A1CM, medium from A1 astrocytes treated with PBM. Representative immunofluorescence images of NeuN (red) and β III tubulin (green) in DRG neurons. Scale bar: 100 mm. **(B,C)** The length of axons in a single neuron and the number of neurons per field were analyzed. Five visual fields from each sample were randomly selected and analyzed. The experiments were independently repeated three times. *p < 0.01; ns, not significant.

A previous study demonstrated A1 astrocytes induce neuronal death by releasing toxic factors (Liddelow et al., 2017). To explore the influence of PBM on the neurotoxicity of A1 astrocytes, we added ACM to cultured DRG neurons and calculated the axon length of single neurons and the number of neurons per visual field to evaluate neuronal survival. Regardless of whether astrocytes in a homeostatic state were exposed to PBM, ACM had no obvious effect on DRG neuron survival; however, when medium from A1 astrocytes (A1CM) was added to DRG neurons, the axons retracted, and the cell number was decreased; these effects were counteracted by PBM (Figures 6A–C). We concluded that PBM alleviates the neurotoxic effects of A1 astrocytes *in vitro*.

## Discussion

The pathological process of SCI can be divided into primary injury and secondary injury according to the pathological stage (Sekhon and Fehlings, 2001). Primary injury is irreversible and refers to insults directly caused by physical injury itself. It is very important to intervene in secondary injury to improve tissue repair and functional recovery. Studies targeting secondary damage have always attracted great interest among experts in neurosurgery, orthopedics, materials science, pharmacology, neurobiology, and rehabilitation physiotherapy; however, due to the complexity of the pathological process of SCI, there are currently no widely recognized therapeutic options (Tran et al., 2018; Courtine and Sofroniew, 2019). As a reliable therapeutic method, PBM refers to “the use of red or NIR to stimulate, heal, regenerate, and protect tissue that has either been injured, is degenerating, or else is at risk of dying” (Hamblin, 2016). Due to its antioxidant and anti-inflammatory effects and its ability to improve blood flow and mitochondrial metabolism in the injured area, PBM has a wide range of applications for PD, Alzheimer’s disease (AD), traumatic brain injury (TBI), ischemic stroke, SCI, and other neurodegenerative diseases (Salehpour et al., 2018). Our team had previously found that 810 nm PBM can inhibit the inflammatory response, reducing the formation of the glial scar, and promoting better motor recovery after SCI (Song et al., 2017; Sun et al., 2020; Zhang et al., 2020).

In this study, we improved the application of PBM by embedding laser fibers into rats subjected to SCI instead of using indirect transcutaneous irradiation to better project the laser to the damaged tissue; the feasibility and safety of this strategy were verified previously (Liang et al., 2019; Zuo et al., 2021). We discovered that PBM induced the transformation of neurotoxic (A1) astrocytes to neuroprotective (A2) astrocytes, which may be a promising strategy for treating SCI, as demonstrated in recent reports (Liu et al., 2019; Qian et al., 2019; Su et al., 2019; Vismara et al., 2020; Zou et al., 2021). RNA-Seq was performed on animal tissues to explore the changes in reactive astrocyte-specific transcript levels in the acute phase of SCI, and representative genes were selected for further verification by RT-PCR. The data showed that A1-specific transcript levels were obviously increased at 7 dpi, A2-specific transcript levels showed a slight increase followed by a decrease until 7 dpi, and PAN-reactive transcript levels basically increased until 14 dpi. This outcome seems to be easy to interpret because A2-specific transcripts were originally defined as a cluster of genes found to be significantly upregulated in astrocytes in an ischemia model, while A1-specific transcripts were defined as those induced by neuroinflammation in an *in vivo* LPS injection model (Zamanian et al., 2012; Liddelow and Barres, 2017). Following physical trauma, vascular disruption and rupture of the blood-spinal cord barrier cause hemorrhage and ischemia in the spinal cord, which may result in the upregulation of A2-specific transcript levels in earlier pathological processes. Subsequently, A1-specific transcript levels were gradually upregulated and reached a peak at 7 dpi due to the inflammatory response, which begins to cause most of the secondary damage (Zamanian et al., 2012; Liddelow and Barres, 2017). However, when A1 astrocytes are activated after SCI is controversial, as Qian et al. (2019) and Jiang et al. (2020) reported that A1 astrocytes can be observed only at 28 dpi, but Zhang Y. et al. (2021) claimed that naïve astrocytes transformed into A1 astrocytes at 3 dpi. These differences may have been due to the unclear definition of A1/A2 astrocyte activation, which was first described (Liddelow et al., 2017) in cell culture conditions. Despite the differences in the conclusions, consistent with other reports, the activation of A1 astrocytes was stronger at 14 dpi than at 7 dpi. For this reason, we hypothesized that A1 astrocyte activation had already occurred to a certain extent at 7 dpi (perhaps even at 3 dpi to some extent) and continued to increase in the following several weeks. Regardless, there is no doubt that intervening with reactive astrocytes within 7 dpi is an excellent strategy because microglia begin to be rapidly recruited to the lesion to play a role and secrete specific inflammatory mediators (TNF-α, IL-1α, and C1q) that are necessary to induce A1 astrocyte activation at the acute phase of SCI (Li T. et al., 2020).

Reactive astrocytes are diverse and have complex functions, and they adopt distinct molecular states in different disease models (Escartin et al., 2021); therefore, we wanted to further probe the mechanism of A1/A2 astrocyte activation in an SCI model. We selected signaling pathways previously reported to be related to A1/A2 activation (not limited to SCI models) and then performed KEGG pathway and GO enrichment analysis. These pathways, including the NF-κB pathway, Notch pathway, JAK2–STAT3 pathway, and PI3K–Akt pathway, were selected for further verification in this study. The results showed that, except for the Notch pathway, which basically showed no obvious change within 7 dpi, the other three pathways began to be promoted after injury and the activation of these pathways was blocked to varying degrees by PBM. We subsequently verified the changes in the four pathways in cultured astrocytes; however, while PBM inhibited A1 astrocyte activation and promoted A2 astrocyte activation, the results were not completely consistent with the *in vivo* results. *In vitro*, the four pathways were found to be activated at a relatively low level both before and after PBM intervention in resting astrocytes. After astrocytes were polarized to the A1 phenotype, the Notch pathway was promoted, but PBM had no effect on it; the activation of other pathways was inhibited by PBM. However, the administration of specific antagonists highlighted that some pathways ultimately exacerbate, alleviate, or have no effect on the activation of astrocytes such that the selective manipulation of one pathway may disguise or secondarily impact the others (Escartin et al., 2021). We confirmed that NF-κB plays a key role in promoting the activation of A1 astrocytes because the expression of C3 was significantly decreased when it was inhibited; additionally, the expression of S100a10 was upregulated when NF-κB was inhibited, which is consistent with previous results (Hou et al., 2020; Liu et al., 2020; Wang et al., 2021). We did not observe obvious changes in the Notch pathway at 7 dpi, although Qian et al. (2019) claimed that the Notch pathway was activated at 28 dpi in rats subjected to SCI. Notch1 expression was upregulated in A1 astrocytes, but PBM had no effect on it, meaning that Notch1 may not be the key target of PBM in regulating astrocyte activation and may only participate in astrocyte activation under culture conditions. The JAK2–STAT3 pathway and PI3K–Akt pathway were both obviously activated after SCI, and PBM downregulated the expression of proteins associated with these pathways, which is consistent with the *in vitro* results. However, when the two pathways were inhibited, the results were different: inhibition of the JAK2–STAT3 pathway led to downregulation of C3 expression, while inhibition of the PI3K–Akt pathway led to upregulation of C3 expression; inhibition of both promoted an increase in S100a10 expression. According to previous studies, the role of STAT3 is complex: evidence has shown that the activation of STAT3 contributes to A1 activation (Zhang L. et al., 2021; Zhang Y. et al., 2021), but some researchers (Ma et al., 2020) have claimed that the phosphorylation of STAT3 is more prone to inducing A2 astrocyte activation. In this study, PI3K–Akt signaling participated in A2 astrocyte activation, which is consistent with the results of Li T. et al. (2020) and Xu et al. (2018). These inconsistent results may be due to the fact that single cells *in vitro* cannot simulate the complex *in vivo* physiological processes well. In addition, PBM has a wide range of targets, and the various signaling pathways interact with each other. Furthermore, a single indicator (C3 or S100a10) can only show part of a variety of integrated effects, which does not demonstrate the elaborate activation of A1/A2 astrocytes.

Of course, in addition to directly acting on astrocytes, PBM may play an indirect role by regulating the level of specific factors. bFGF and TGF-β attracted our attention because the expression of both was upregulated by PBM treatment after SCI. Under culture conditions, regardless of whether astrocytes were induced, PBM promoted the release of bFGF and TGF-β, and this phenomenon was reversed by specific inhibitors. When recombinant proteins were added to the astrocyte medium, both had a similar ability to upregulate A2-specific transcript levels but did not significantly change PAN-reactive transcript levels or A1-specific transcript levels in resting astrocytes at certain concentrations. When bFGF and TGF-β were added to A1 astrocytes 24 h after induction at the same concentrations, the upregulation of PAN-reactive transcript levels and A1-specific transcript levels was inhibited, and A2-specific transcript levels were increased. We concluded that the dose-dependent upregulation of these transcript levels in cultured astrocytes induced by the addition of bFGF and TGF-β had a similar effect as PBM in regulating A1/A2 astrocyte transformation. bFGF has important pleiotropic effects in astrocytes under various physiological and pathological conditions and controls the complex morphology of reactive astrocytes (Fogarty et al., 2005; Irmady et al., 2011; Agarwal and Bergles, 2014; Kang et al., 2014). TGF-β was also proven to downregulate the expression of A1-specific markers and to alleviate the neurotoxic response in induced activated astrocytes (Gottipati et al., 2020). The increase in the levels of bFGF and TGF-β induced by PBM treatment explained the ability of PBM to promote the transformation of astrocyte phenotype to some extent. Interestingly, both factors were also reported to promote SCI repair by other means (Logan and Berry, 1993; Zhang et al., 2013; Jahan and Hannila, 2015; Ye et al., 2016). Finally, we confirmed that the change in reactive astrocyte-specific transcript levels was consistent with the distinctive influence on DRG neurons and that neurotoxicity of A1 astrocytes was alleviated by PBM treatment. Combined with our previous conclusions regarding the mechanisms underlying PBM treatment, including the ability of PBM to reduce oxidative stress and secondary inflammation, regulate the polarization of M1/M2 macrophages, inhibit glial scar formation, and promote axon regeneration (Song et al., 2017; Sun et al., 2020; Zhang et al., 2020), our current results led us to hypothesize that these phenomena are interrelated and participate in the therapeutic effect of PBM on SCI. However, we were unable to determine the proportion of reactive astrocytes of each phenotype involved in this process and whether it is the main mechanism or one of multiple similar mechanisms.

**FIGURE 7:**
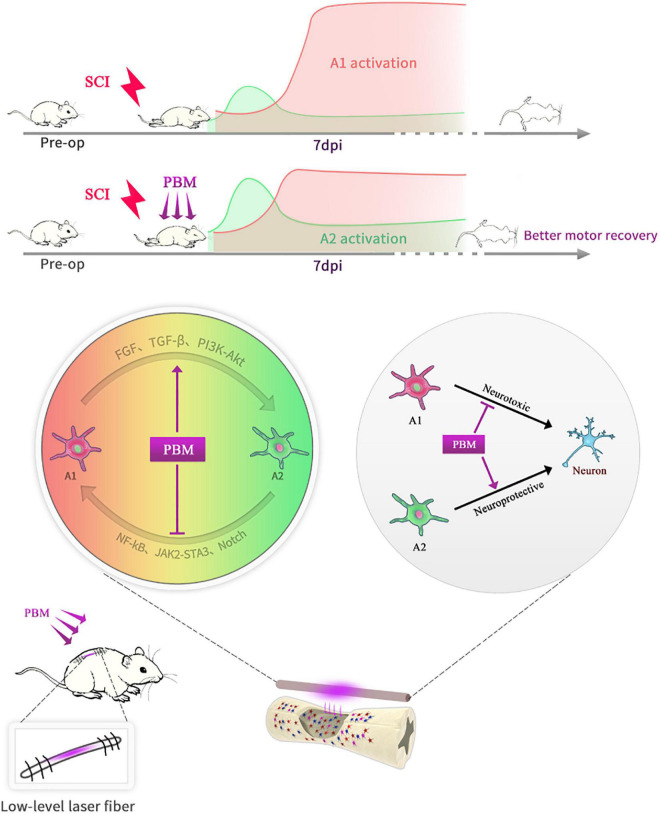
Schematic diagram showing that PBM regulates the transformation of A1/A2 reactive astrocytes. An implantable fiber was embedded in the rat vertebral plate after SCI, and PBM improved motor recovery and promoted tissue repair. At 7 dpi, A1 astrocyte activation was suppressed, and A2 astrocyte activation was promoted after PBM treatment. The mechanism may be related to the ability of PBM to reduce the toxicity of A1 astrocytes to neurons by altering the activation of specific signaling pathways and factors associated with A1/A2 astrocyte activation.

In short, as shown in the schematic diagram (Figure 7), PBM can improve motor recovery and promote tissue repair after SCI, partly by regulating the phenotypic transformation of reactive astrocytes in both animal models and under *in vitro* conditions. The underlying mechanism is manifold. On the one hand, PBM modulates signaling pathways related to astrocyte activation; on the other hand, the levels of some cytokines, such as bFGF and TGF-β, are changed by PBM, and these factors indirectly affect astrocyte activation. There are some deficiencies in this study: we did not explore the relationships among these signaling pathways or the mechanisms by which bFGF and TGF-β were stimulated by PBM. In addition, astrocytes have long been more involved in the process of glial scar formation, which hinders axon regeneration. We did not further explore the relationship between this process and the phenotypic transformation of A1/A2 astrocytes; however, this topic should be explored further in the future. Furthermore, only male rats were used in this study and our findings cannot be extrapolated to females without further research. We will continue to explore the mechanism by which PBM exerts its biological effects and the mechanism of astrocyte activation and phenotypic transition.

## Data Availability Statement

The original contributions presented in the study are publicly available. This data can be found here: National Center for Biotechnology Information (NCBI) BioProject database under accession number PRJNA760277.

## Ethics Statement

The animal study was reviewed and approved by the Institutional Animal Care and Use Committee of Air Force Military Medical University.

## Author Contributions

XW, XH, and ZW designed and conceived the study. XW, ZZhu, and ZL established the SCI model and performed the PBM. ZZha, ZZhu, XZ, CJ, ZS, and XL participated in the *in vitro* experiments. XW and ZZhu performed the analysis for all assays. XW wrote the manuscript. XH and ZW revised the manuscript. All authors contributed to the article and approved the submitted version.

## Conflict of Interest

The authors declare that the research was conducted in the absence of any commercial or financial relationships that could be construed as a potential conflict of interest.

## Publisher’s Note

All claims expressed in this article are solely those of the authors and do not necessarily represent those of their affiliated organizations, or those of the publisher, the editors and the reviewers. Any product that may be evaluated in this article, or claim that may be made by its manufacturer, is not guaranteed or endorsed by the publisher.
